# A Literal Interlineal Translation of the First Ten Chapters of Gregory's Conspectus Medicinæ Theoreticæ; with the Original Text. To Which Are Added, an Ordo Verborum, and Rules for Construing, &c.

**Published:** 1836-04

**Authors:** 


Art. XI.—*A Literal Interlineal Translation of the first Ten Chapters of Gregory's Conspectus Medicinæ Theoreticæ; with the original Text. To which are added, an Ordo Verborum, and Rules for Construing, &c. &c.* By Robert Venables, a.m. m.b. Oxon.—*London*, 1836. 12mo. pp. 122.

Applying the same remarks to the introductory matter of the present little work which we made respecting that of Mr. Underwood, just noticed, as being utterly inadequate to the teaching of the Latin language to those who are ignorant of it, (if, indeed, this is contemplated by the author, who certainly does not formally say so,) we are bound to state, that a partial inspection of the text in various parts of it leads us to believe that the translation is, on the whole, very correct and judicious, and cannot fail to be very useful to those who are sincerely desirous of following up their studies of the Latin medical classics. We agree with the author in thinking that a translation of a work from a language of which we are commencing the study, or rather, we should say, in which we have already made a little progress, is, if well executed, calculated to assist the industrious, while it can scarcely make the idle worse than they were before: but we need hardly say, after what we have stated in the preceding Notice, that the student who fondly imagines that the best and most literal translation will stand him instead of a thorough elementary knowledge of grammar, acquired in the usual way, by long and painful study, will sooner or later find himself utterly and miserably deceived.

And here we would venture to suggest to the Examiners at Apothecaries' Hall, the propriety of varying from time to time the books submitted to the candidates for licences as tests of their classical knowledge. If it is once generally known—and the multitude of recent works similar to that now before us seems to countenance the belief, (nay, we are told that in some of them the fact is boldly announced,) that the examinations are invariably confined to a certain number of books of Celsus or Gregory, it will require, we imagine, no very superior ingenuity for a youth very indifferently skilled in Latin, by the aid of such translations as that now before us, and the terrible assiduity of a two months' drill under an experienced leader, to exhibit a very complete mastery, as far as rendering into English goes, of all the pages submitted to him on the day of examination. If, in place of this, no particular books were fixed on as the subject of examination, or if the announced list was enlarged so as to take in some three or four more authors,—say, for instance, Mead, Heberden, Sir G. Baker, Lommius, or any other *easy* yet good writers, (for we by no means wish to make the examination a severe one,) *the cramming* "for the nonce," which we have mentioned as practicable at least, if not practised, could hardly take place.

